# Identification of differentially expressed genes to predict the risk of heart failure in older patients with hypertrophic cardiomyopathy

**DOI:** 10.18632/aging.205956

**Published:** 2024-06-20

**Authors:** Hao Dong, Chufan Yin, Dongping Xiao, Yong Tang

**Affiliations:** 1 Department of Cardiology, The Second Hospital of Nanjing, Nanjing University of Chinese Medicine, Nanjing 210000, China; 2 Education Section, The Second Hospital of Nanjing, Nanjing University of Chinese Medicine, Nanjing 210000, China; 3 Department of Cardiology, The First Hospital of Nanchang, Nanchang 330000, China

**Keywords:** hypertrophic cardiomyopathy, bioinformatics, microarray, heart failure, differential expressed gene

## Abstract

Aim: Hypertrophic cardiomyopathy (HCM) is a common heart disease. Old people with HCM are at high risk of heart failure (HF). This study aimed to identify differentially expressed genes (DEGs) to evaluate the risk of HF in older patients with HCM.

Methods: GSE89714 and GSE116250 were downloaded from Gene Expression Omnibus (GEO) database, and DEGs were identified by using limma R package with *P* < 0.05 and logFC> 1 as cut off. Protein-protein interaction (PPI) network, Genome Ontology (GO) and Kyoto Encyclopedia of Genes and Genomes (KEGG) analyses were performed for the identified DEGs. NetworkAnalyst online tool was applied for Gene Set Enrichment Analysis (GSEA) analysis.

Results: We identified 124 overlap DEGs from the 2 datasets. PPI network showed that COL1A1, COL3A1, COL1A2, BGN, COL5A1, LUM, TGFB2, FMOD, ASPN, and COL14A1 were the top ten genes related to HCM and HF compared with control. Functional and pathway analyses showed that the overlap genes were mainly related to ECM-receptor interaction, ECM organization, Focal adhesion, PI3K-Akt signaling, TGF-beta signaling, and Platelet activation signaling and aggregation. Among the overlap genes, COL5A1 and LUM were significantly upregulated, while TGFB2, FMOD, ASPN, and COL14A1 were significantly downregulated in HF dataset compared with HCM dataset.

Conclusions: Bioinformatics-based analysis revealed potential genes associated with HCM and HF, which could be utilized to evaluate the risk of HF in older patients with HCM.

## INTRODUCTION

Hypertrophic cardiomyopathy (HCM) is a common heart disease characterized by the hypertrophy of left ventricle (LV) [1]. Older patients with HCM have a high risk of heart failure (HF) and sudden cardiac death (SCD) [2].

The development of HF in HCM patients remains unclear [3]. In most patients with HCM, HF shows the phenotype of HF with preserved ejection fraction (HFpEF), but few patients with HCM develop HF with reduced ejection fraction (HFrEF) at a late stage [4]. Several studies showed that the prevalence of HF in patients with HCM could be from 50% to 67% [5, 6]. The high morbidity and mortality of older HCM patients indicate that it is important to investigate the mechanism by which HCM patients develop HF.

The development of bioinformatics tools to explore gene expression data provides the opportunity to understand the molecular mechanism of a variety of diseases [7, 8]. This study aimed to identify differentially expressed genes (DEGs) to evaluate the risk of HF in older patients with HCM based on bioinformatics analysis of gene expression data in patients with HCM and HF.

## MATERIALS AND METHODS

### High-throughput sequencing data and differentially expressed genes analysis

The high-throughput sequencing datasets GSE89714 and GSE116250 were downloaded from Gene Expression Omnibus (GEO) database. GSE89714 dataset formed the study of “Differential gene expressions in the heart of hypertrophic cardiomyopathy patients”, and GSE116250 formed the study of “RNA-seq of heart failure in human left ventricles”. The DEGs were analyzed using linear models for microarray analysis R program with *P* < 0.05 and logFC > 1 as cut off. The heatmap and the hub genes were created using the NetworkAnalyst program (https://www.networkanalyst.ca/) [9].

### Network establishment

The overlap of HCM and HF datasets’ DEGs were outlined with Venn diagram (https://bioinfogp.cnb.csic.es/tools/venny/index.html). HCM-overlap genes-HF network was established using Cytoscape 3.8.0 software, and protein-protein interactions (PPIs) of the DEGs were analyzed using STRING database (https://string-db.org/).

### Gene functional and pathway enrichment analysis

Gene Ontology (GO) and Kyoto Encyclopedia of Genes and Genomes (KEGG) enrichment analyses were performed using the Database for Annotation, Visualization, and Integrated Discovery (DAVID) database (https://david.ncifcrf.gov/). NetworkAnalyst online tool was applied for Gene Set Enrichment Analysis (GSEA) analysis. Briefly, GO items for the overlap genes were collected and imported into GSEA software to identify significantly enriched GO items.

### Statistical analysis

Data were analyzed by using GraphPad Prism 8.0 software. Two-tailed Student’s t-test was used to compare the data in two groups. *P* < 0.05 was deemed significant.

### Availability of data and materials

All data are available from the corresponding author on request.

## RESULTS

### Identification of DEGs in HCM and HF patients

**Figure 1:**
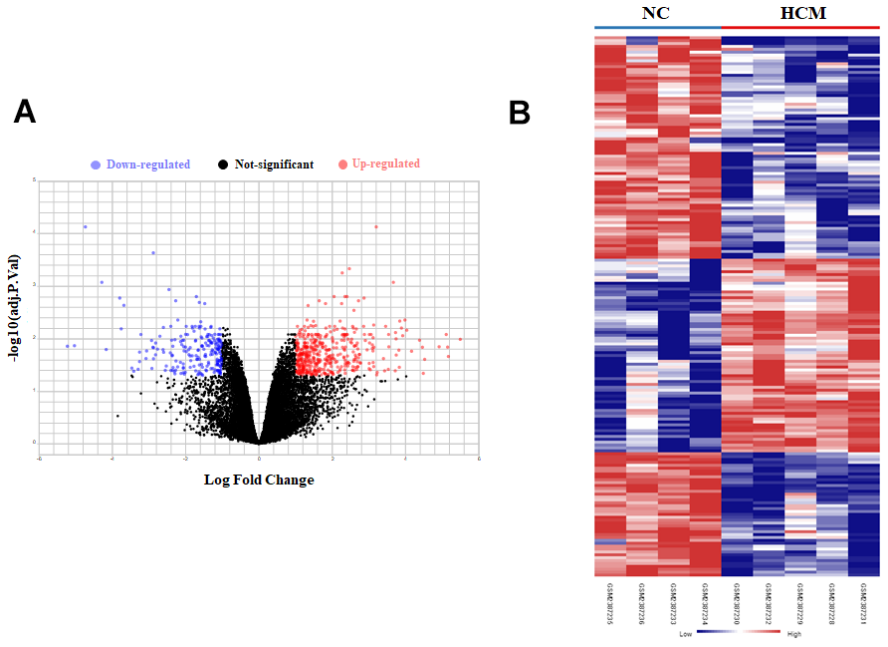
**Bioinformatic analysis of DEGs in LV tissue of normal control (NC) and hypertrophic cardiomyopathy (HCM) patients.** (**A**) The volcano plot of DEGs in LV tissue between NC group and HCM group. (**B**) Heatmaps of DEGs in LV tissue of NC group and HCM group. Red color indicated high expression while blue color indicated low expression.

**Figure 2:**
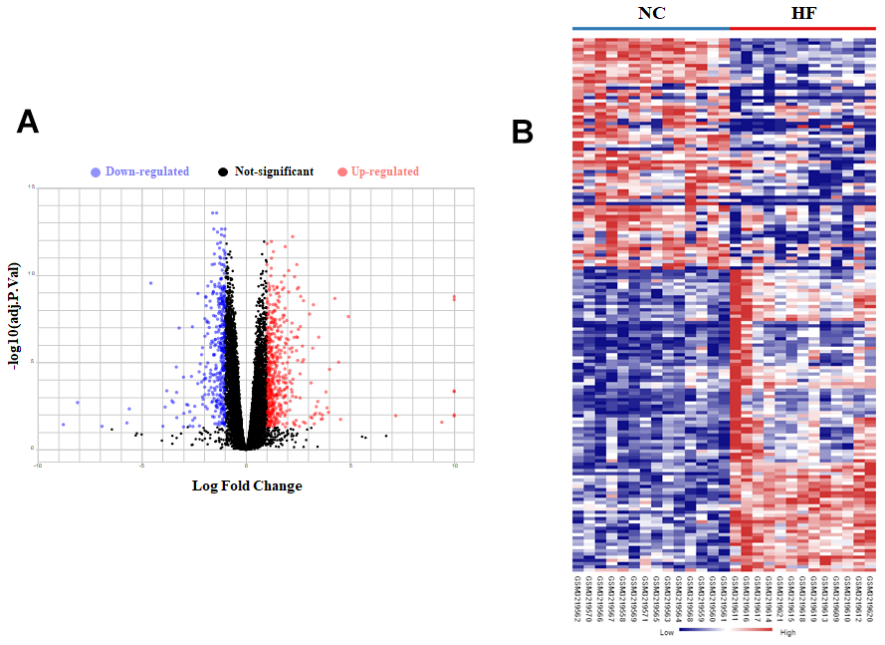
**Bioinformatic analysis of DEGs in LV tissue of normal control (NC) and heart failure (HF) patients.** (**A**) The volcano plot of DEGs in LV tissue between NC group and HF group. (**B**) Heatmaps of DEGs in LV tissue of NC group and HF group. Red color indicated high expression while blue color indicated low expression.

LV tissues high-throughput sequencing data datasets GSE89714 and GSE116250 were downloaded from GEO database. The volcano plot and heatmap of DEGs showed a total of 655 DEGs with 437 upregulated and 218 downregulated in HCM patients (Figure 1), and a total of 859 DEGs with 490 upregulated and 369 downregulated in HF patients (Figure 2).

### The protein-protein interaction analysis of the HCM and HF overlap genes

**Figure 3:**
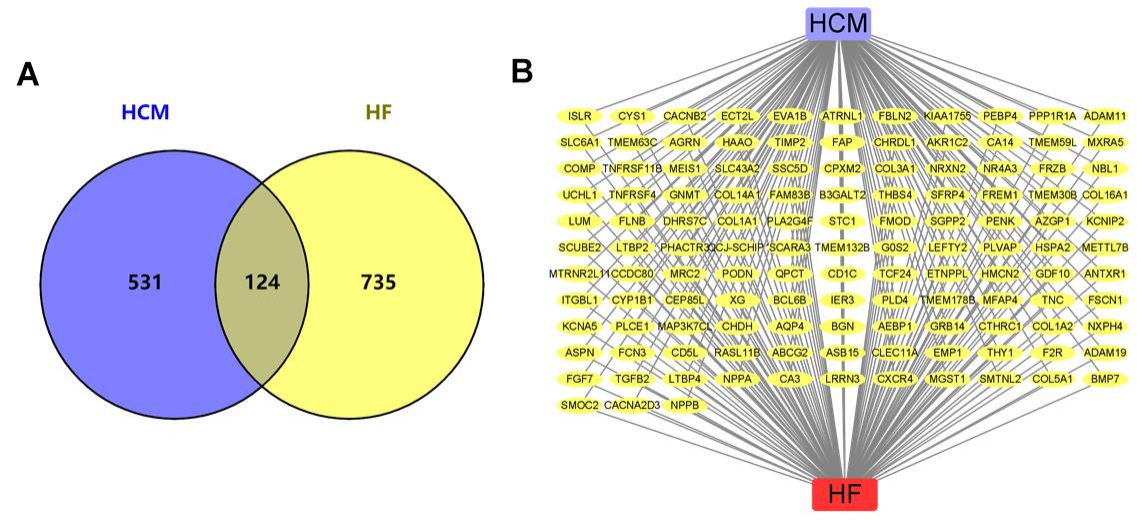
**HCM and HF datasets overlap DEGs.** (**A**) Venn diagram of HCM and HF DEGs. (**B**) The network of HCM, HF, and all the overlap DEGs. Yellow nodes represented the overlap DEGs.

To further analyze the relationship between HCM and HF, the interaction of HCM and HF DEGs was shown in a Venn diagram. We found 124 genes closely related to HF progression (Figure 3A). In addition, we established an HCM-overlap gene-HF network (Figure 3B), highlighting that HCM may deteriorate to HF through these genes.

**Figure 4:**
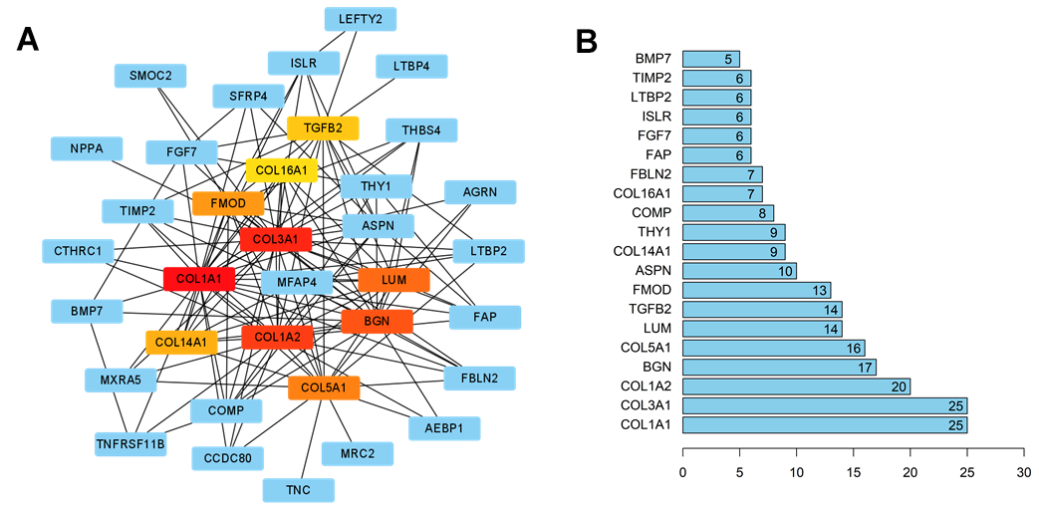
**The interaction network of HCM and HF overlap genes.** (**A**) PPI network of the overlap genes. The hub genes were represented as red and yellow nodes. A deeper red color indicated more connections. (**B**) Bar plot of the number of hub gene links.

PPI analysis of the 124 overlap genes established a network: the top 10 hub genes were highlighted in red, and other genes connected with hub genes were shown as blue nodes (Figure 4A). Hub genes such as COL1A1, COL3A1, COL1A2, BGN, and COL5A1 are involved in the biological process of HF (Figure 4B).

### Functional enrichment analysis of overlap genes

**Figure 5:**
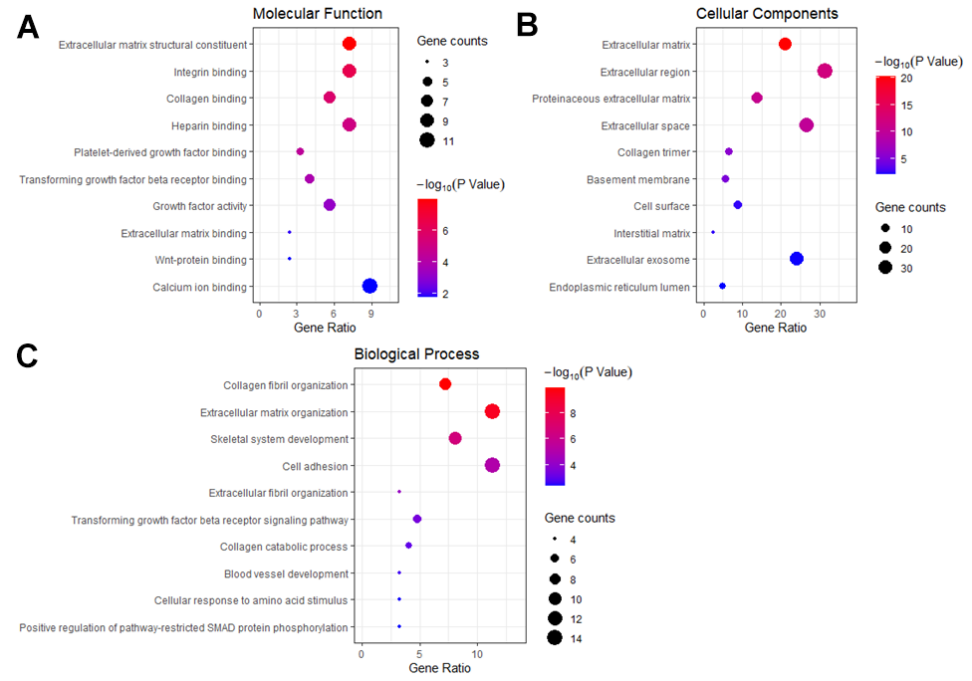
**GO enrichment analysis of HCM and HF overlap genes.** (**A**) The plot of enriched molecular functions. (**B**) The plot of enriched cellular components. (**C**) The plot of enriched biological processes. The number of genes enriched in each GO term was shown as the circle size, and the p-value was shown as different colors.

GO enrichment analysis showed that extracellular matrix (ECM) structural constituent and collagen binding (Figure 5A), ECM and extracellular region (Figure 5B), and collagen fibril organization and ECM organization (Figure 5C) were enriched and could play an important role in the biological process of HF.

**Figure 6:**
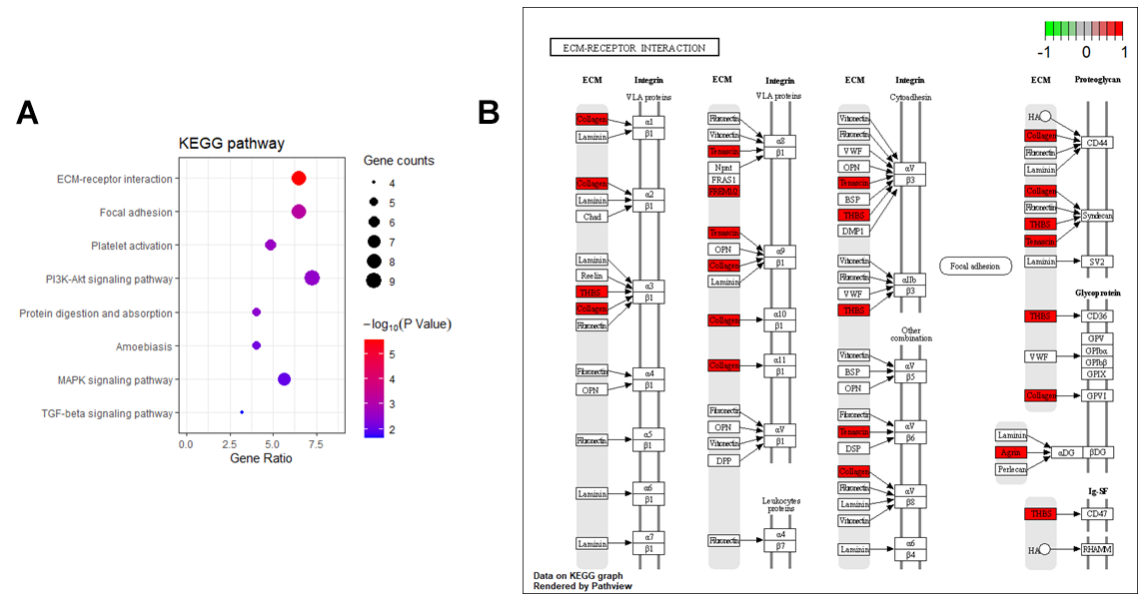
**KEGG enrichment analysis of the HCM and HF overlap genes.** (**A**) KEGG annotation of overlap genes. The number of genes enriched in each KEGG term was shown as the circle size, and the p-value was shown as different colors. (**B**) The overlap genes were mainly distributed in the ECM-receptor interaction pathway. Arrows represented activation effect, T-arrows represented inhibition effect and segments showed activation effect or inhibition effect. The red nodes were the intersection genes.

**Figure 7:**
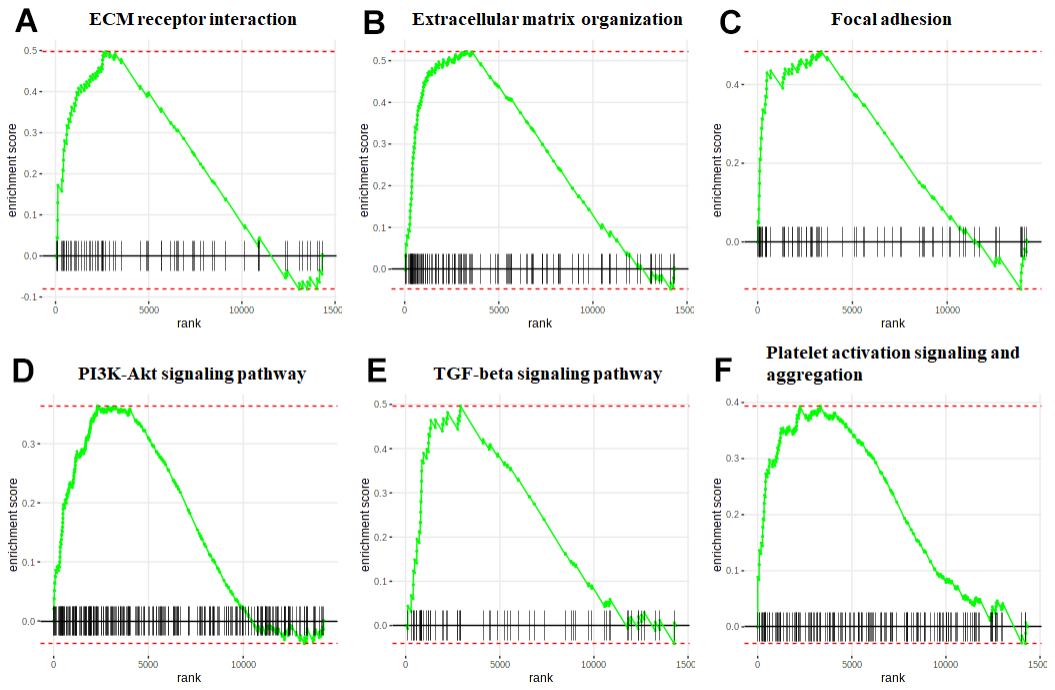
**GSEA of the HCM and HF overlap genes.** The enrichment plot of the ECM receptor interaction (**A**), Extracellular matrix organization (**B**), Focal adhesion (**C**), PI3K-Akt signaling pathway (**D**), TGF-beta signaling pathway (**E**), and Platelet activation signaling and aggregation (**F**). The green curve represented the enrichment profile, and the black vertical line represented the gene hits.

In addition, eight KEGG pathways were enriched (Figure 6A) with genes mainly distributed in ECM-receptor interaction pathway (Figure 6B). Furthermore, GSEA analysis of the overlap genes showed that the DEGs were significantly correlated with ECM organization, ECM receptor interaction, Focal adhesion, TGF-beta signaling, PI3K-Akt signaling, and Platelet activation signaling and aggregation (Figure 7).

### The changes of overlap hub gene expression in HCM and HF datasets

**Figure 8:**
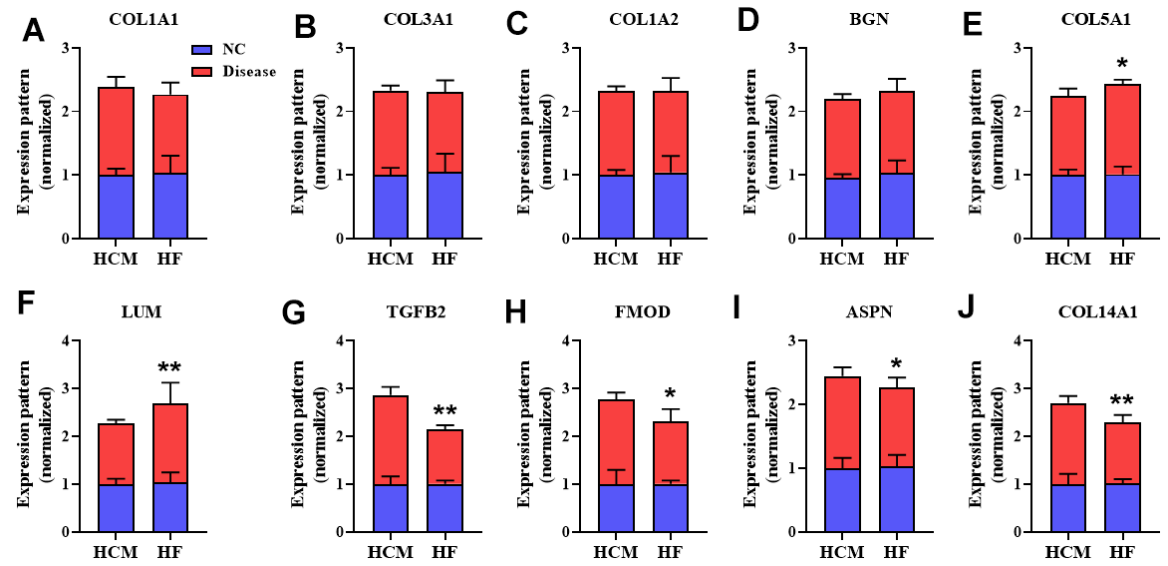
**The overlap genes expression pattern in HCM and HF datasets.** The expression pattern of COL1A1 (**A**), COL3A1 (**B**), COL1A2 (**C**), BGN (**D**), COL5A1 (**E**), and LUM (**F**), TGFB2 (**G**), FMOD (**H**), ASPN (**I**), COL14A1 (**J**) in different datasets. Values were normalized to NC group and represented as Mean ± SD (*n* = 3 in HCM dataset, *n* = 13 in HF dataset). ^*^P < 0.05, ^**^P< 0.01 vs. HCM disease group.

Finally, we selected ten hub genes COL1A1, COL3A1, COL1A2, BGN, COL5A1, LUM, TGFB2, FMOD, ASPN, and COL14A1 to confirm their expression patterns in HCM and HF datasets (Figure 8). Among them, COL5A1 and LUM were significantly increased in the HF dataset (*P* < 0.05, Figure 8E, 8F), but TGFB2, FMOD, ASPN, and COL14A1 were significantly decreased in the HF dataset (*P* < 0.05, Figure 8G–8J).

## DISCUSSION

The rate of HF complications in HCM patients ranges from 2.4% to 20%, and increases significantly in older patients [10, 11]. However, the mechanistic link between HCM and HF remains unclear. Therefore, in this study we utilized gene expression datasets of HCM and HF to identify 124 overlap genes. Among them, top ten hub genes included COL1A1, COL3A1, COL1A2, BGN, COL5A1, LUM, TGFB2, FMOD, ASPN, and COL14A1. These results revealed that DEGs were mainly involved in myocardial fibrosis, and HCM may deteriorate to HF through these genes.

Myocardial fibrosis in pathological cardiac remodeling contributes to HCM and HF [12]. COL5A1 is an alpha chain for one of the low abundance fibrillar collagens, and regulates the function of connective tissues [13]. As a member of the leucine-rich proteoglycan family, LUM regulates the assembly of collagen fibers, and both COL1A1 and LUM are involved in heart fibrosis [14].

To gain insight into the biological function of the overlap genes, we performed GO enrichment analysis. The overlap genes were enriched in the following functional categories including, ECM structural constituent and Collagen binding, cellular components as ECM and Extracellular region, and molecular function as Collagen fibril organization and ECM organization. KEGG pathway analysis showed that the DEGs were enriched in ECM organization, ECM receptor interaction, Focal adhesion, TGF-beta signaling, PI3K-Akt signaling, and Platelet activation signaling and aggregation. ECM-receptor interaction and Focal adhesion have been shown to be important for HCM and HF progression [15]. A previous study showed that platelet aggregation was positively correlated with LV hypertrophy [16]. TGFβ signaling is known to play a role in hypertrophy [17]. Notably, a recent study identified six TGFβ related genes involved in both cardiac hypertrophy and HF based on single-cell RNA sequencing [18].

The limitations of this study should be pointed out. This study is *in silico* and our results should be confirmed by further *in vivo* studies. Moreover, we only screened the DEGs and pathways involved in HCM and HF. Further dissection of these DEGs and pathways will help elucidate the mechanism underlying the progression of HCM and HF.

In conclusion, we performed bioinformatics analysis to identify potential targets involved in HCM and HF, which could be utilized to evaluate the risk of HF in older patients with HCM.
